# Neferine Protects Endothelial Glycocalyx via Mitochondrial ROS in Lipopolysaccharide-Induced Acute Respiratory Distress Syndrome

**DOI:** 10.3389/fphys.2018.00102

**Published:** 2018-02-22

**Authors:** Xiang-Yong Liu, Hai-Xiao Xu, Jian-Kui Li, Dong Zhang, Xiao-Hong Ma, Li-Na Huang, Jun-Hong Lü, Xiao-Zhi Wang

**Affiliations:** ^1^Department of Cell Biology, Binzhou Medical University, Yantai, China; ^2^Department of Respirator Medicine and Intensive Care Unit, Affiliated Hospital of Binzhou Medical University, Binzhou, China; ^3^Division of Physical Biology and CAS Key Laboratory of Interfacial Physics and Technology, Shanghai Institute of Applied Physics, Chinese Academy of Sciences, Shanghai, China

**Keywords:** acute respiratory distress syndrome, neferine, endothelial glycocalyx, lipopolysaccharide, mitochondrial ROS

## Abstract

Damage to the endothelial glycocalyx is a critical factor in increased pulmonary vascular permeability, which is the basic pathological feature of acute respiratory distress syndrome (ARDS). Neferine (Nef), a bisbenzylisoquinoline alkaloid isolated from green seed embryos of *Nelumbo nucifera Gaertn*, has extensive pharmacological activity. In this study, we showed that Nef reduced lung-capillary permeability, down-regulated the production of cytokines (IL-1β, IL-6, TNF-α, and IL-10) and inhibited the activation of the NF-κB signaling pathway in mice with lipopolysaccharide (LPS)-induced ARDS. Further analysis indicated that Nef provided protection against endothelial glycocalyx degradation in LPS-induced ARDS mice (*in vivo*) and in LPS-stimulated human umbilical vein endothelial cells (*in vitro*). The glycocalyx-protective effect of Nef may be initiated by suppressing the production of mitochondrial ROS (mtROS) and decreasing oxidative damage. Nef was also found to promote glycocalyx restoration by accelerating the removal of mtROS in endothelial cells in LPS-induced ARDS. These results suggested the potential of Nef as a therapeutic agent for ARDS associated with Gram-negative bacterial infections and elucidated the mechanisms underlying the protection and restoration of the endothelial glycocalyx.

## Introduction

Acute respiratory distress syndrome (ARDS), a significant source of morbidity and mortality in critically ill patients, is characterized by the abrupt onset of clinically significant hypoxemia with the presence of diffuse pulmonary infiltrates. An increase in pulmonary vascular permeability is the basic pathological feature of ARDS, which can further lead to the efflux of protein-rich edema fluid into the pulmonary alveolus, impairing gas exchange across the alveolar membrane and ultimately causing respiratory failure (Ware and Matthay, 2000; Wheeler and Bernard, 2007; Laffey and Kavanagh, 2017; Smith et al., 2017).

The endothelial glycocalyx is a network of membrane-bound proteoglycans and glycosaminoglycans that covers the healthy vascular endothelium (Yang and Schmidt, 2013). It mostly consists of a core protein and side chains. The core protein mostly comprises proteoglycans, such as syndecan-1 (SDC-1). The side chains consist of glycosaminoglycans, such as heparin sulfate (HS). The endothelial glycocalyx serves as the primary physical barrier and interface between the blood and the vessel wall and plays a crucial role in regulating vascular endothelial permeability (Salmon and Satchell, 2012; Yang and Schmidt, 2013; Mehta et al., 2014; Chelazzi et al., 2015). It also plays an important role in the mechanosensing that mediates the NO production that protects endothelial cells from blood-flow-induced shear stress. The endothelial glycocalyx also modulates inflammatory responses by mediating adhesion of circulating inflammatory cells to the endothelium and by binding cytokines and thus attenuating the binding of inflammatory cytokines to cell surface receptors. The endothelial glycocalyx is also involved in antioxidant defense and anticoagulant processes by binding antioxidative enzyme (e.g., superoxide dismutase) and anticoagulant mediators (e.g., antithrombin III). Damage to the glycocalyx can have many pathophysiological consequences, such as increased vascular permeability, edema formation, increased adhesion of circulating inflammatory cells to the endothelium, accelerated inflammatory process, activation of the coagulation cascade, and platelet hyperaggregation. (Reitsma et al., 2007; Yang and Schmidt, 2013; Kolárová et al., 2014; Chelazzi et al., 2015; Yen et al., 2015; Schött et al., 2016; Sieve et al., 2018).

In addition to ARDS, the endothelial glycocalyx has been demonstrated to play an important role in several diseases, such as acute kidney injury (Libório et al., 2015), diabetes (Dogné et al., 2016), ischemia/reperfusion (van Golen et al., 2014), and hemorrhagic shock (Naumann et al., 2017). Therefore, approaches to protect against glycocalyx damage and promote glycocalyx restoration have an important clinical significance (Martin et al., 2016; Yang et al., 2017).

Neferine (Nef), a bisbenzylisoquinoline alkaloid isolated from green seed embryos of *Nelumbo nucifera* Gaertn, has showed therapeutic effects in several diseases and exhibits anti-tumor, anti-oxidative, anti-inflammatory, anti-fibrosis, anti-arrhythmic, and anti-platelet effects (Jung et al., 2010; Zhao et al., 2010; Peng et al., 2011; Zhou et al., 2013; Baskaran et al., 2016; Eid and Abdel-Rehim, 2017; Sivalingam et al., 2017). Previous reported showed neferine could attenuate bleomycin-induced pulmonary fibrosis by suppressing inflammatory responses and oxidative stress (Zhao et al., 2010). It also could inhibit human lung cancer cell growth and potentiates the anti-lung cancer effect of cisplatin by inducing apoptosis (Poornima et al., 2014; Sivalingam et al., 2017). However, there now little has been known about its effect on ARDS.

In this study, we determined the effect of Nef on endothelial glycocalyx protection/restoration and its underlying mechanisms in lipopolysaccharide (LPS)-induced ARDS. The results of the study provided valuable information for drug development of endothelial glycocalyx as a therapeutic target and on the regulatory mechanisms of endothelial glycocalyx protection/restoration.

## Materials and methods

### Chemicals and antibodies

Nef was purchased from Shanghai Ziqi Biology Technology Co., Ltd. (Shanghai, China), with purity of 99.16%. Mouse polyclonal to heparan sulfate antibody was purchased from AMS Biotechnology (Europe) (Switzerland, MA). Rabbit monoclonal to Syndecan-1 was purchased from Abcam (Cambridge, USA). Thrombomodulin /BDCA-3 was purchased from R&D Systems (Minnesota, USA). Fluorescein isothiocyanate (FITC)-conjugated anti-goat IgG and rhodamine-conjugated anti-mouse IgG were purchased from Zhongshan Golden Bridge Biotechnology (Beijing, China).

### Animals and grouping

All experiments were conducted in accordance with the National Institute of Health Guide for the Care and Use of Laboratory Animals (NIH Publication No. 80–23; revised in 1996) and were approved by the Animal Care and Use Committee of Binzhou Medical University. C57BL/6 mice (male, 8–10 weeks old) were purchased from Jinan Pengyue Laboratory Animal Center (Shandong, China).

**Figure 1:**
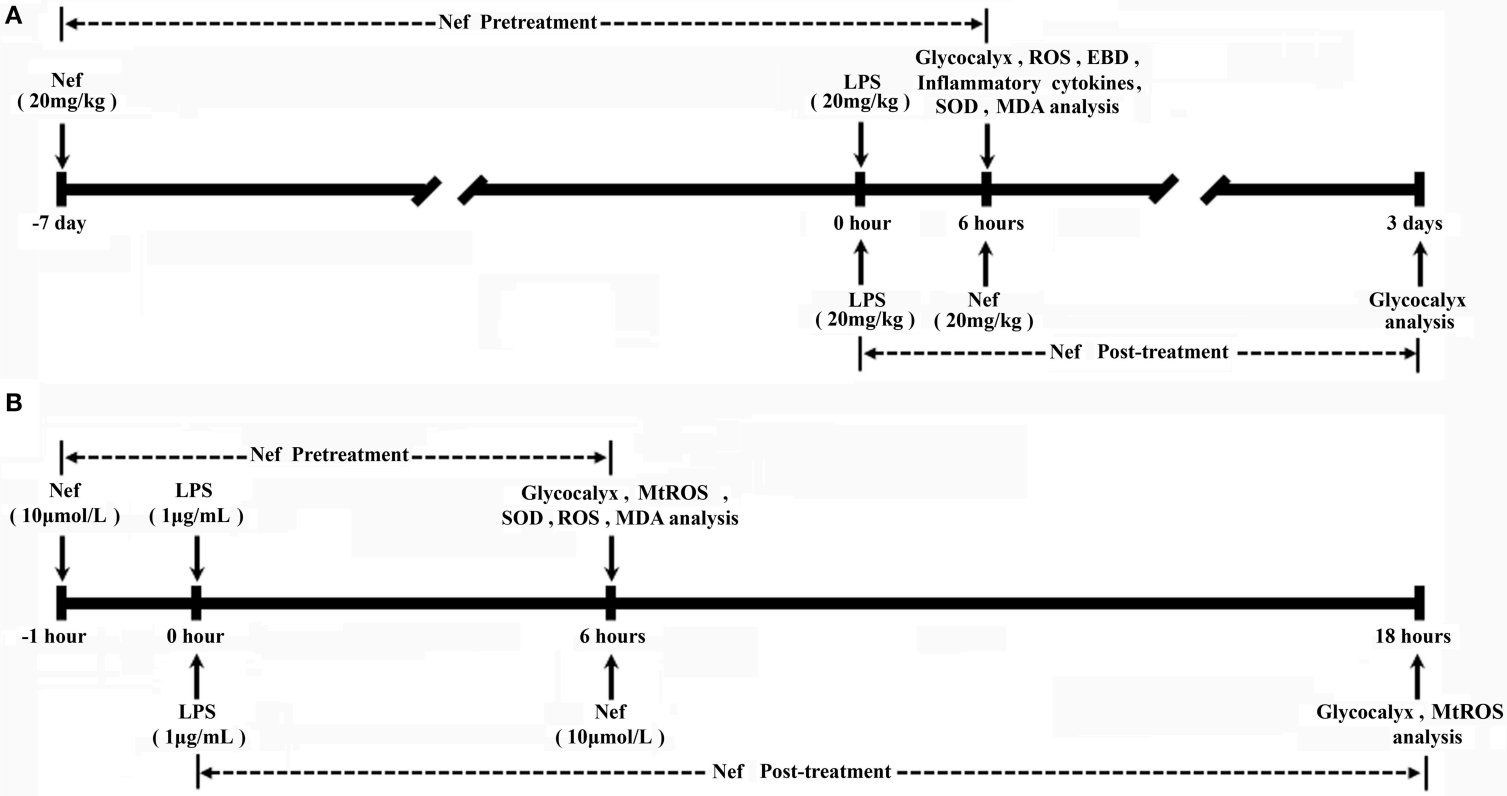
Experimental schedule. **(A)** Nef pretreatment: Mice mice were gavaged with a single dose of Nef solution (20 mg/kg/day) for 7 days, then were intraperitoneally injected with LPS (20 mg/kg) LPS to induce ARDS. Nef post-treatment: At 6 h after LPS administration, mice were gavaged with a single dose of Nef solution (20 mg/kg/day) for 3 days. **(B)** Nef pretreatment: HUVECs were pretreated with 10 μM Nef for 1 h, followed by LPS (1 μg/mL) stimulation for 6 h. Nef post-treatment: HUVECs were treated with 10 μM Nef for 12 h after they were treated with LPS (1 μg/mL) LPS for 6 h.

Grouping for analyzing the effect of Nef pre-treatment in protecting GCX shedding: Experimental mice were randomly allocated into control, LPS, Nef + LPS, and Nef groups. Mice in the Nef + LPS and Nef groups were gavaged with a single dose of Nef solution (20 mg/kg/day) for 7 days, and mice in the control and LPS groups received equal volume of normal saline instead of Nef in the same manner. Subsequently, the Nef + LPS and LPS groups were intraperitoneally injected with LPS (20 mg/kg) to induce ARDS as previously reported (Ma et al., 2015). The control and Nef groups received an equal volume of normal saline without LPS. At 6 h after LPS administration, mice lung tissue, bronchoalveolar lavage fluid, and serum samples were collected for further analysis. Grouping for analyzing the effect of neferine on Nef post-treatment in promoting GCX restoration: Experimental mice were randomly allocated into the control, LPS, Nef-repairing, and self-repairing groups. The LPS, Nef-repairing, and self-repairing groups were intraperitoneally injected with LPS (20 mg/kg) to induce ARDS. The control received an equal volume of normal saline without LPS. At 6 h after LPS administration, the mice in the LPS group were killed immediately for analysis, those in the Nef-repairing group were gavaged with a single dose of Nef solution (20 mg/kg/day) for 3 days, and those in the control and self-repairing groups received an equal volume of normal saline without Nef. After the above treatments, the mice were euthanized with intraperitoneal injection of 4% Chloral hydrate, then the chests were opened and the lungs were excised by blunt dissection for further analysis. The time schedule of those experiments was shown in Figure 1A.

### Cell culture and treatment

Human umbilical vein endothelial cells (HUVECs) were grown to confluence and cultured in 25 cm^2^ flasks supplemented with complete culture medium (AllCells, Shanghai, China) in a CO_2_ incubator with humidified atmosphere of 95% air and 5% CO_2_ at 37°C. Cells at 80–90% confluence were used for all assays, which were then then plated at an appropriate density according to each experimental scale.

Grouping for analyzing the effect of Nef pre-treatment in protecting GCX shedding: Control cells were not exposed to LPS. LPS group: HUVECs were treated with LPS (1 μg/mL) for 6 h. Nef + LPS group: HUVECs were pretreated with 10 μM Nef for 1 h followed by LPS (1 μg/mL) stimulation for 6 h. Nef group: HUVECs were treated with 10 μM Nef for 7 h. Grouping for analyzing the effect of Nef post-treatment in promoting GCX restoration: Control cells were not exposed to LPS. LPS group: HUVECs were treated with LPS (1 μg/mL) for 6 h. Self-repairing groups: the culture medium of HUVECs was replaced with complete medium without LPS after treatment with LPS (1 μg/mL) for 6 h. Nef-repairing group: the culture medium of HUVECs was replaced with complete medium containing 10 μM Nef after treatment with LPS (1 μg/mL) for 6 h. The time schedule of those experiments was shown in Figure 1B.

### Histopathology and lung injury score

Lung tissues were excised and washed with saline solution. They were then fixed in 4% formalin and embedded in paraffin. They were cut into 5 μm sections, stained with hematoxylin and eosin (H&E), and visualized under an optical microscope (Olympus Optical, Tokyo, Japan) for histological analysis. Tissue sections were then evaluated based on the method described by (Wang L. et al., 2016), which using the following scoring system to grade the degree of lung injury. Features were focal thickening of the alveolar membranes, congestion, pulmonary edema, intra-alveolar hemorrhage, interstitial neutrophil infiltration, and intra-alveolar neutrophil infiltration. Each feature was assigned a score from 0 to 3 based on its absence (0) or presence to a mild (1), moderate (2), or severe (3) degree, and the total cumulative histology score was determined.

### Wet/dry lung weight ratio

The lung was excised, blotted dry, weighed to obtain the wet weight, and then placed in an oven at 80°C for 72 h to obtain the dry weight. The wet/dry (W/D) ratio was used as an indicator of tissue edema.

### Pulmonary permeability assay

Evans blue (EB) dye extravasation technique was used to determine lung-capillary permeability. At the end of LPS exposure (*Escherichia coli*, 055:B5, Sigma-Aldrich), EB (20 mg/kg, Sigma-Aldrich) was administered via the tail vein. Circulation continued for 30 min. Lung vasculature was perfused with 20 mL of saline through the spontaneously beating right ventricle. Lungs harvested from each mice were placed in formamide (Sinopharm Chemical Reagent Co., Ltd, Shanghai, China) to extract EB for 24 h as described previously. The extraction liquid was centrifuged at 12,000 × g. Dye content was evaluated at 620 nm absorption with a microplate reader. Lung EB level was calculated against a standard curve and expressed as ng EB dye/mg lung.

### Cell viability assay

The effect of differential concentrations of Nef on the cell viability of HUVECs was evaluated using 3-4,5-dimethylthiazol-2-yl-2,5-diphenyltetrazolium bromide (MTT) assay and determining the ratios of cells undergoing Nef-induced apoptosis and necrosis. MTT assay: briefly, 4 × 10^5^ cells were seeded in each well of a 96-well plate and allowed for attachment overnight. Cells were then incubated with Nef in 0, 5, 10, 20, 30 and 40 μM concentrations for 18 h. Subsequently, 10 μL of MTT (5 mg/mL) solution was added to each well of the 96-well plate, and incubation continued for 4 h. After 4 h, the supernatant was removed, and the formation of formazan was resolved with 150 μL/well of DMSO. The absorbance was measured at 570 nm using a microplate absorbance reader (SpectraMax M2, Molecular Devices). The Nef-induced apoptosis/necrosis ratio was evaluated using an Apoptosis and Necrosis Assay Kit (Beyotime, China) according to the manufacturer's instructions. Briefly, the cells were added to 24-well culture plates with 1 × 10^6^ cells per well and treated with 0, 5, 10, 20, 30, and 40 μM Nef for 18 h. Subsequently, the cells were washed twice with PBS and then stained with Hoechst 33342 and propidium iodide (PI) for 30 min at 4°C in the dark. Next, the stained cells were washed with PBS and then observed using a fluorescence microscope (Olympus BX53, Tokyo, Japan). More than 500 random selected cells were measured under each condition.

### Western blot analysis

At 6 h after LPS intraperitoneal injection, the lung tissues were harvested, homogenized and stored at −80°C until use. The extraction of nuclear and cytoplasmic proteins from the lung tissues was performed using a nuclear and cytoplasmic protein extraction kit (Beyotime, China) in accordance with the manufacturer's protocol. The total protein from the lung tissues was lysed in radioimmune precipitation assay (RIPA) buffer supplemented with protein phosphatase inhibitor (Solarbio, China). The protein concentrations were determined using a BCA protein assay kit, and proteins were loaded into each well on a 10% SDS polyacrylamide gel. The proteins were then transferred onto polyvinylidene difluoride membranes (PVDF). These membranes were washed in Tris-buffered saline with Tween 20 (TBST) and incubated in 7% skim milk for 2 h at room temperature to reduce non-specific binding and washed again in TBST. Subsequently, the samples were probed overnight at 4°C with primary antibodies, including NF-κB/P65 (ab32536, Abcam) and phosphorylated forms of IkB-α (ab133462, Abcam) and nonphosphorylated forms of IkB- α (ab32518, Abcam). The mixture was then diluted in 1:1000 primary antibody diluent. Lamin B1(ab133741, Abcam) and GAPDH (10494-1-AP, proteintech) were performed as an internal control for nuclear and cytoplasmic protein loading, respectively. The membranes were then washed with TBST followed by incubation with peroxidase-conjugated secondary antibody at room temperature for 1 h. Protein bands were detected using an ECL detection kit (Thermo Fisher Scientific, Inc.) in accordance with the manufacturer's instructions.

### BALF collection and measurement of cytokines

After 6 h of LPS treatment, bronchoalveolar lavage fluid (BALF) was collected three times through a tracheal cannula with autoclaved PBS that was instilled up to a total volume of 1.5 mL. The BALF samples were immediately centrifuged (4°C, 3,000 rpm, 10 min). Levels of IL-1β, IL-6, TNF-α, and IL-10 in the BALF were evaluated with the corresponding ELISA kits (Beyotime and Neobioscience, China) in accordance with the manufacturer's instructions.

### Immunofluorescence

To detect the levels of lung endothelial HS and SDC-1, 4 μm paraffin-embedded lung sections were cut, deparaffinized sequentially, blocked with goat or rabbit serum blocking solution (Solarbio, Beijing, China), and incubated with mouse polyclonal to heparan sulfate antibody (1:100, United States Biological, Swampscott, MA) or rabbit monoclonal to Syndecan-1 (1:463, ab128936, Abcam) overnight at 4°C. Sequentially, the sections were washed three times with PBS and incubated with rhodamine-conjugated anti-mouse IgG (1:100, ZSGB-BIO, Beijing, China) for 1 h at room temperature. The sections were then washed three times with PBS and incubated with the secondary antibody goat polyclonal to thrombomodulin/BDCA-3 (1:100, AF3894, R&D Systems) overnight at 4 °C. Sequentially, the sections were washed three times with PBS and incubated with FITC-conjugated anti-goat IgG (1:100, ZSGB-BIO, Beijing, China) for 1 h at room temperature. The sections were then washed three times with PBS and incubated with 4′,6-diamidino-2-phenylindole dihydrochloride (DAPI; Sigma-Aldrich) for 8 min at room temperature. Finally, the sections were washed three times with PBS and blocked with fluorescence decay-resistant medium (Solarbio, Beijing, China). Sections were visualized using a microscope (Olympus BX53, Tokyo, Japan). The relative quantitative processing of data was performed via Image J software, and the results were statistically analyzed by SPSS 17.0.

To detect the levels of HUVECs endothelial HS and SDC-1, cells were seeded on coverslips in 24-well plates. HUVECs were treated with Nef for 1 h followed by LPS for 6 h. After LPS exposure, cells were washed three times with PBS and fixed with 4% paraformaldehyde for 15 min at room temperature. Subsequently, the cells were washed in PBS three times and then incubated directly in goat serum blocking solution. They were then incubated with antibodies to HS and SDC-1 overnight at 4°C. Primary antibody binding was detected using FITC-conjugated and rhodamine-conjugated secondary antibodies. Subsequently, the nuclei were stained with DAPI. The relative quantitative processing of data was performed via Image J software, and the results were statistically analyzed by SPSS 17.0.

### Measurement of HS /SDC-1 in mouse serum and HUVECs culture medium

The levels of shed HS and SDC-1 in mouse serum and HUVEC culture medium were determined using corresponding ELISA kits. A Human HS ELISA Kit and mouse HS ELISA kit were purchased from Mlbio, China. A Human SDC-1 ELISA Kit and Mouse SDC-1 ELISA Kit were purchased from Diaclone, France.

### Assay of ROS level in HUVECs and mice lung tissue

The production of cytoplasmic ROS in HUVECs was monitored using a non-fluorescent probe, 2′,7′-dichlorofluorescein diacetate (DCFH-DA, Sigma-Aldrich, USA). DCFH-DA passively diffuses into cells and is deacetylated, changing into the fluorescent compound, 2′,7′-dichlorofluorescein (DCFH). DCFH reacts with ROS to form the fluorescent product, DCF, which is trapped inside the cells. A total of 1 × 10^5^ cells were seeded on coverslips in 24-well plates and allowed for attachment overnight. In total, 1 mM of *N*-acetyl cysteine (NAC) served as a positive control for the generation of intracellular ROS in HUVECs. HUVECs were treated with Nef for 1 h followed by LPS for 6 h. After LPS exposure, DCFH-DA, diluted to a final concentration of 10 μM with basal culture medium (AllCells, Shanghai, China), was added to HUVECs followed by incubation for 20 min at 37°C and protected from light. After incubation, HUVECs were washed three times with PBS. DCF fluorescence was then assayed using a fluorescence microscope (Olympus BX53, Tokyo, Japan). Finally, the data were presented as relative fluorescence intensity values of intracellular ROS.

ROS generation in lung tissue was measured as described previously with slight modification. Briefly, 190 μL tissue homogenates were placed in 96-well plates and then incubated with 10 μL DCFH-DA (1 mM) for 30 min at 37 °C and protected from light. After 30 min incubation, the conversion of DCFH-DA to the fluorescent product DCF was measured using a spectrofluorimeter (SpectraMax M2, Molecular Devices) with excitation at 484 nm and emission at 530 nm. ROS formation was quantified using a DCF standard curve, and data were expressed as μmol DCF formed/min/mg protein.

### Measurement of mitochondrial ROS production

Mitochondrial ROS (mtROS) detection studies were performed using MitoSOX™ Red mitochondrial superoxide indicator (M36008, Invitrogen). MitoSOX™ Red mitochondrial superoxide indicator is a fluorogenic dye for highly selective detection of superoxide in the mitochondria of live cells. A total of 1 × 10^5^ cells were seeded on coverslips in 24-well plates and allowed for attachment overnight. In total, 50 μM of Mito-TEMPO served as a positive control for the generation of mtROS in HUVECs. HUVECs were treated with Nef for 1 h followed by LPS for 6 h. After LPS exposure, the cells were incubated with 5 μM MitoSOX™ Red for 10 min at 37°C and protected from light. After incubation, they were washed thrice gently with PBS buffer to remove excess unbound dye and replaced with fresh basal culture medium. The production of mtROS was measured using a fluorescence microscope (Olympus BX53, Tokyo, Japan). Finally, the data were presented as relative fluorescence intensity values of mtROS.

### Measurement of malondialdehyde content and superoxide dismutase activity

The lung was excised, placed immediately in cold saline, homogenized in cold phosphate buffer, and centrifuged at 1,600 × g for 10 min at 4°C. Supernatants were used to assess the concentration of malondialdehyde (MDA) and superoxide dismutase (SOD) activity via commercial kits following the manufacturer's instructions (Beyotime, China). Protein concentrations were measured by bicinchoninic acid (BCA) protein assay. Lung MDA concentration was expressed as nmol/mg protein and SOD activity was expressed as U/mg protein.

A total of 1 × 10^6^ cells were seeded on six-well plates and allowed for attachment overnight. HUVECs were treated with Nef for 1 h followed by LPS for 6 h. They were homogenized in cold phosphate buffer and centrifuged at 1,600 × g for 10 min at 4°C. Supernatants were used to determine MDA concentration via commercial kits following the manufacturer's instructions (Beyotime, China). Protein concentrations were measured by BCA protein assay. HUVECs MDA concentration was expressed as nmol/mg protein and SOD activity was expressed as U/mg protein.

### Statistical analysis

The data were shown as the mean ± standard deviation (SD). Statistical differences were assessed using Student's *t*-test and one-way ANOVA followed by the SNK test for multigroup comparison; statistical significance was considered at *p* < 0.05.

## Results

### Nef reduced lung-capillary permeability in LPS-induced ARDS

**Figure 2:**
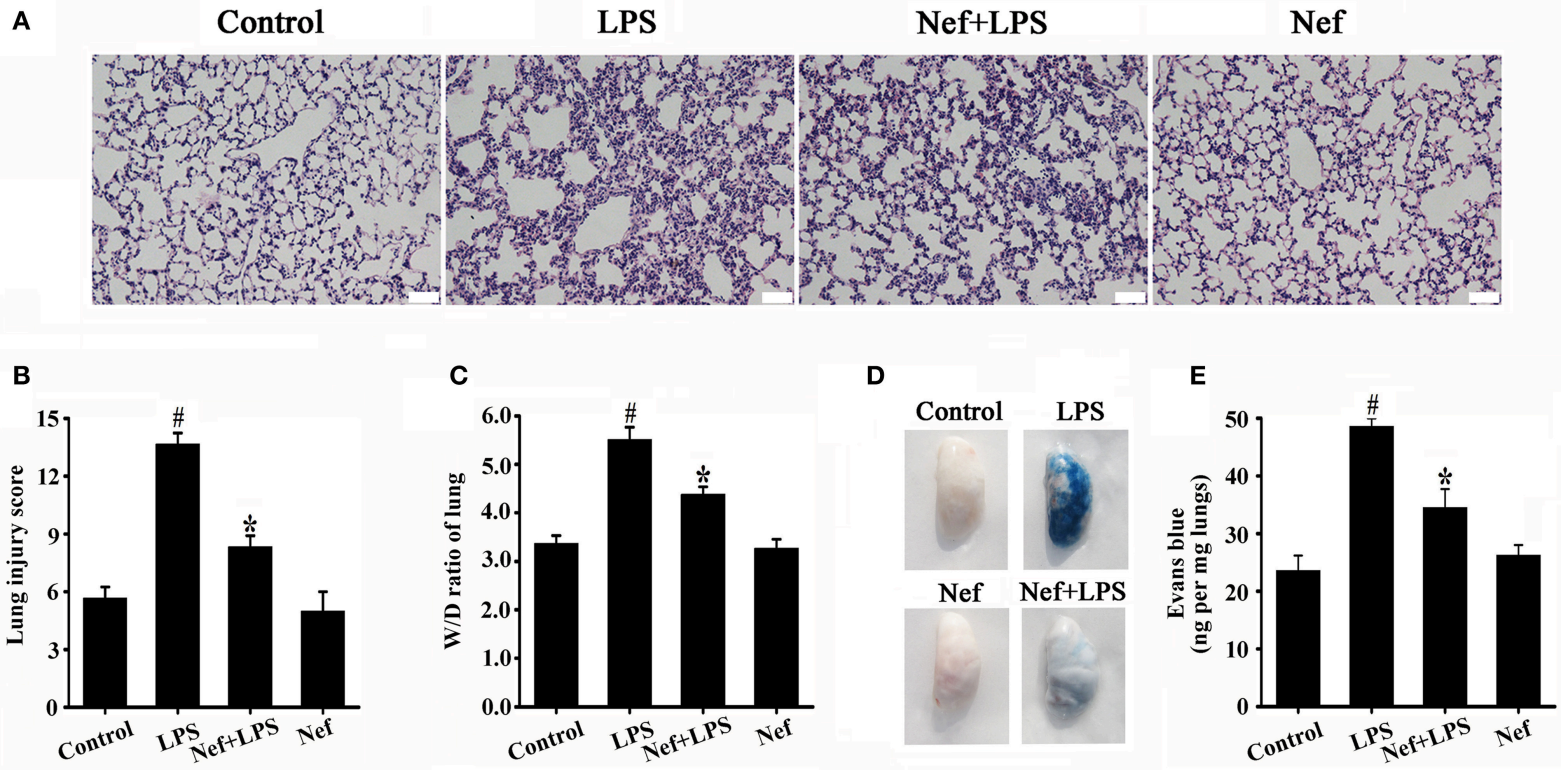
Effect of Nef on histopathologic changes, lung W/D ratio, and lung-capillary permeability in LPS-induced ARDS. C57BL/6 male mice were gavaged with Nef (20 mg/kg/day) for 7 days followed by LPS (20 mg/kg) for 6 h. The lung tissue was collected for analysis of histopathologic changes by H&E staining (*n* = 6 mice/group; magnification 200×, scale bar: 50 μm) **(A,B)**, and lung W/D ratio **(C)**. After LPS exposure, EB was administered via the tail vein, and the values of EB in lung tissues were measured (*n* = 8 mice/group) **(D) (E)**. Data were presented as mean ± SD for three independent experiments. ^#^*p* < 0.05 compared with the control group; ^*^p < 0.05 compared with the LPS group.

The increase in pulmonary vascular permeability is the basic pathological feature of ARDS. We investigated the effect of Nef on the pulmonary vascular permeability in LPS-induced ARDS. We found that Nef pretreatment significantly inhibit the infiltration of inflammatory cells, the thickening of alveolar walls, and the diapedesis of red blood cells (Figures 2A,B). In addition, Nef also reduces the levels of lung W/D ratio (Figure 2C). Furthermore, lung permeability analysis showed that Nef intervention can ameliorate the value of EB leakage into the lung tissue compared with that in the LPS group (Figures 2D,E), suggesting an inhibition effect of Nef on lung-capillary permeability.

### Nef decreased the production of proinflammatory cytokine and inhibited Nf-κB activation in LPS-induced ARDS

**Figure 3:**
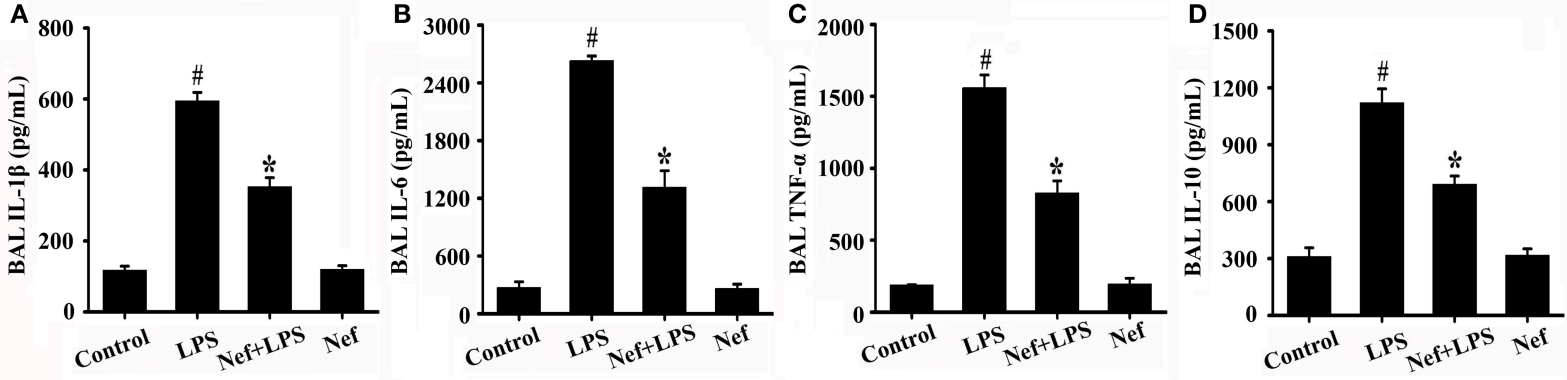
Effect of Nef on IL-1β, IL-6, TNF-α, and IL-10 production in the BALF of LPS-induced ARDS mice. BALF were collected from mice that were gavaged with 20 mg/kg/day Nef for 7 days, followed by 20 mg/kg LPS for 6 h. The levels of IL-1β **(A)**, IL-6 **(B)**, TNF-α **(C)**, and IL-10 **(D)** in BALF were measured using the ELISA kits. Data were presented as mean ± SD for three independent experiments. ^#^*p* < 0.05 compared with the control group; ^*^p < 0.05 compared with the LPS group.

**Figure 4:**
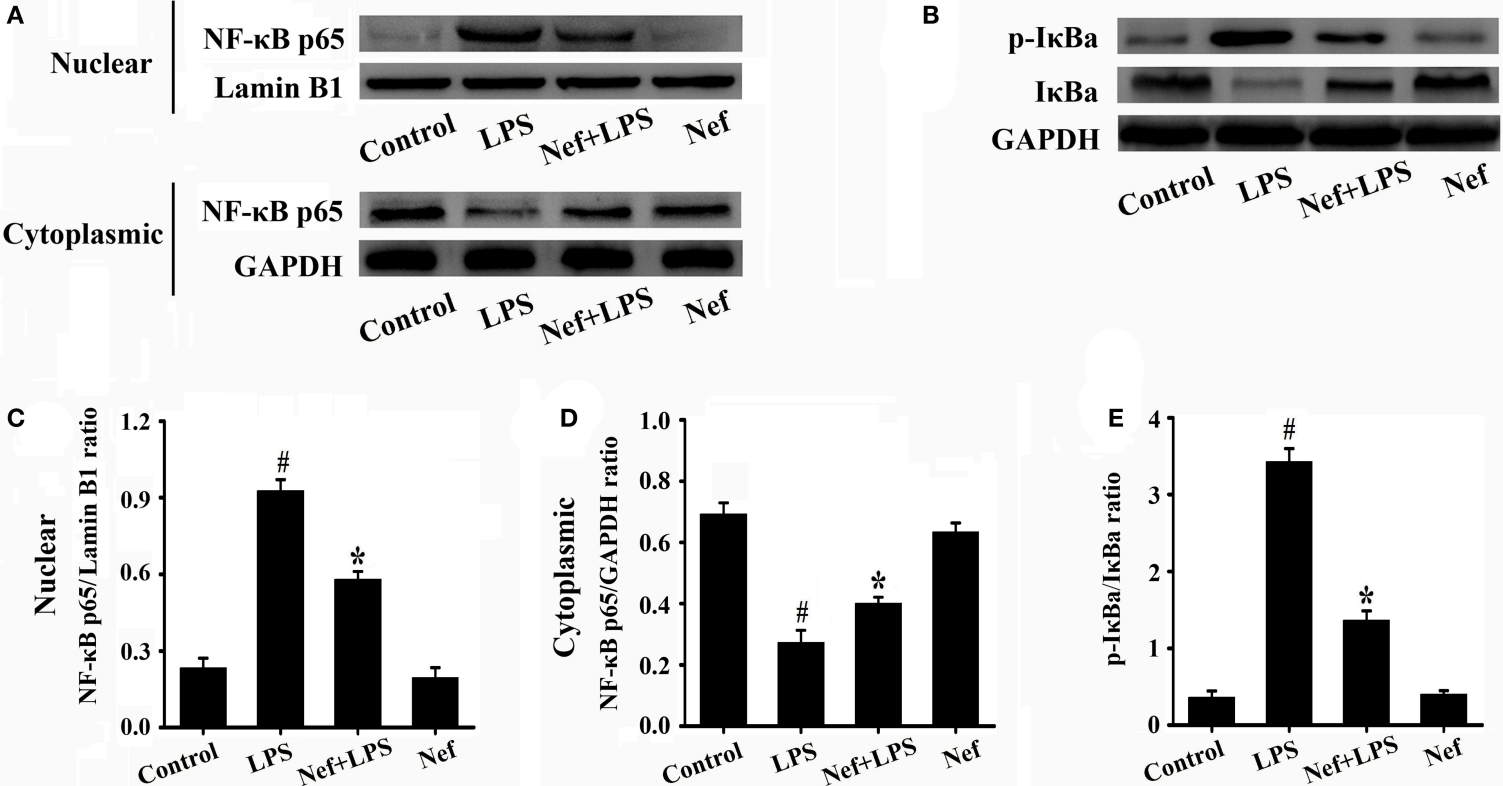
Effect of Nef on NF-κB activation in LPS-induced ARDS mice. Lung tissues were harvested from mice gavaged with 20 mg/kg/day of Nef for 7 days and then with 20 mg/kg of LPS for 6 h (*n* = 6 mice/group). The nuclear translocation levels of NF-κB p65 **(A)** and phospho-IκBα **(B)** in the lung tissues lysates were determined by Western blot. **(C)** Ratio of nuclear NF-κB p65 to Lamin B1 of **(A)**. **(D)** Ratio of cytoplasmic NF-κB p65 to GAPDH of **(A)**. **(E)** Ratio of phospho-IκBα to IκBα **(B)**. Data were presented as mean ± SD for three independent experiments. ^#^*p* < 0.05 compared with the control group; ^*^p < 0.05 compared with the LPS group.

Inflammatory responses play a critical role in the onset and development of ARDS. The effects of Nef on IL-1β, IL-6, TNF-α, and IL-10 production in the BALF of LPS-induced ARDS mice were measured. As shown in Figure 3, treatment with Nef downregulated the levels IL-1β, IL-6, TNF-α, and IL-10, rendering them lower than those in the LPS group. The NF-κB signal pathway plays an important role in the regulation of inflammatory processes (Tak and Firestein, 2001; Zhou et al., 2014). In this way, we analyzed the effects of Nef on NF-κB activation. As shown in Figure 4, after LPS stimulation, the level of nuclear translocation of NF-κB p65 and phospho-IκBα in lung tissues became markedly higher than in the control group. Pretreatment with Nef inhibited NF-κB p65 translocation (Figures 4A,C,D) and the phosphorylation of IκBα (Figures 4B,E). Taken together, these results suggested that Nef could ameliorate inflammatory responses in LPS-induced ARDS.

### Nef protected against endothelial glycocalyx degradation in LPS-induced ARDS

**Figure 5:**
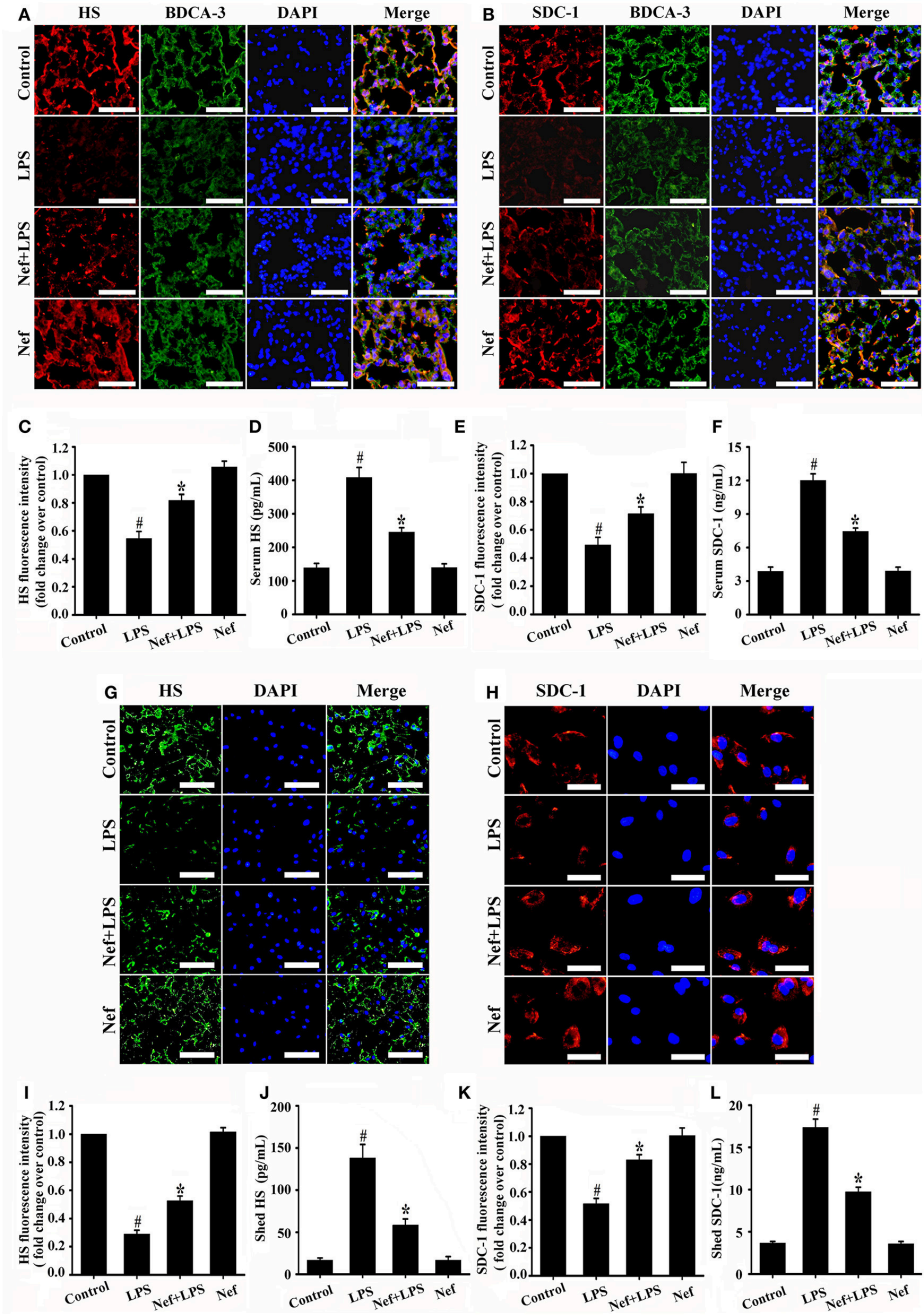
Effect of Nef on endothelial glycocalyx degradation in LPS-induced ARDS. Mice were treated with LPS (20 mg/kg) for 6 h, pretreated with Nef (20 mg/kg/day) for 7 days followed by LPS (20 mg/kg) for 6 h, or pretreated with Nef (20 mg/kg/day) alone for 7 days (*n* = 6 mice/group). The content of HS (red) **(A)** and SDC-1 (red) **(B)** in mice lungs were determined by detected by immunofluorescence. Thrombomodulin / BDCA-3 was used as an endothelial cell marker. (magnification 200×, scale bar: 50 μm). **(C,E)** Fluorescence intensity analyses of **(A,B)**, respectively. The serum levels of HS **(D)** and SDC-1 **(F)** in mice were detected by ELISA kits. HUVECs were treated with LPS (1 μg/mL) for 6 h, Nef (10 μM) for 1 h followed by LPS (1 μg/mL) for 6 h, or Nef (10 μM) alone for 7 h. The content of HS (green, magnification 100×, scale bar: 100 μm) **(G)** and SDC-1 (red, magnification 200×, scale bar: 50 μm) **(H)** in HUVECs detected by immunofluorescence. **(I,K)** Fluorescence intensity analyses of **(G,H)**, respectively. The levels of shed HS **(J)** and SDC-1 **(L)** in HUVECs culture medium were determined by ELISA kits. Data were presented as mean ± SD for three independent experiments. #*p* < 0.05 compared with the control group; ^*^p < 0.05 compared with the LPS group.

Endothelial glycocalyx plays a crucial role in regulating vascular permeability of ARDS. The changes in main endothelial glycocalyx compositions (HS and SDC-1) were commonly used to evaluate glycocalyx integrity (Schmidt et al., 2012; Peng et al., 2013; Zeng et al., 2014; Han et al., 2016). Consistent with previous studies, LPS simulation could causes significant shedding of HS and SDC-1 in both *in vivo* ARDS mice model (Figures 5 A–F) and *in vitro* HUVECs model (Figures 5 G–L), Nef pretreatment significantly suppresses the shedding of HS (Figures 5A,D,G,J) and SDC-1 (Figures 5B,F,H,L) either in ARDS mice or in HUVECs. However, Nef treatment alone showed no effect on the glycocalyx compositions (HS and SDC-1) in mice (Figures 5A,B) and has no toxicity to HUVECs from 0 μM to 20 μM in the MTT assay (Figure S1). These results suggested that Nef can protect against endothelial glycocalyx degradation in LPS-induced ARDS *in vivo* and vitro, which maybe contribute to decrease lung-capillary permeability.

### Nef can promoted endothelial glycocalyx restoration in LPS-induced ARDS

**Figure 6:**
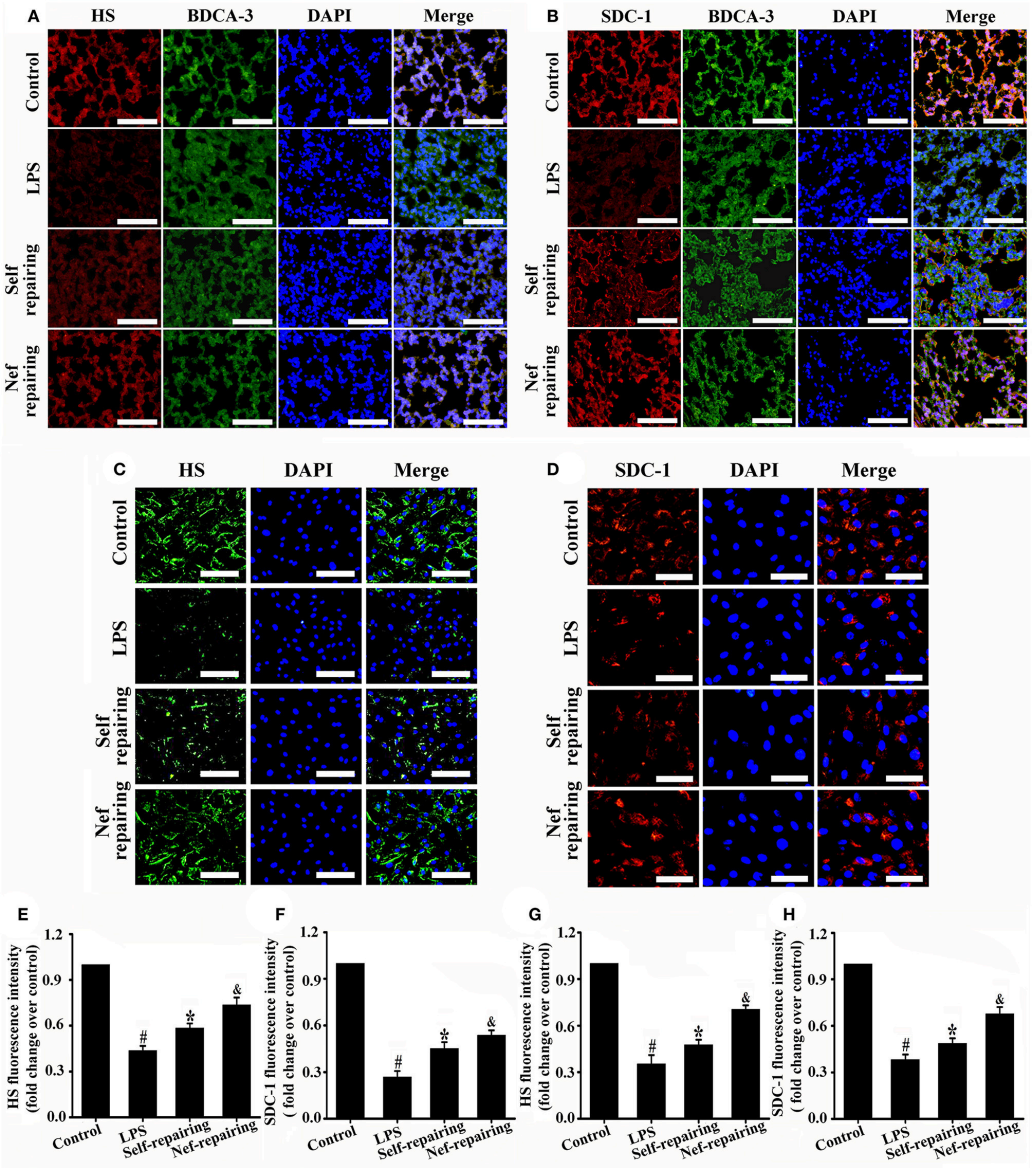
Effect of Nef on endothelial glycocalyx restoration in LPS-induced ARDS. Representative immunofluorescence images of HS component (red) **(A)** and SDC-1 component (red) **(B)** in control mice lungs and those treated with LPS (20 mg/kg) for 6 h, LPS (20 mg/kg) for 6 h followed by normal saline for 3 days, or LPS (20 mg/kg) for 6 h followed by Nef (20 mg/kg/day) treatment for 3 days (n = 6 mice/group, magnification 200×, scale bar: 50 μm). Thrombomodulin/BDCA-3 was used as endothelial cell marker. Representative immunofluorescence images of HS component (green, magnification 100×, scale bar: 100 μm) **(C)** and SDC-1 component (red, magnification 200×, scale bar: 50 μm) **(D)** in control HUVECs and those treated with LPS (1 μg/mL) for 6 h, LPS (1 μg/mL) for 6 h followed by complete medium without LPS for 12 h, or LPS (1 μg/mL) for 6 h followed by Nef (10 μM) for 12 h. **(E–H)** Fluorescence intensity analyses of **(A–D)**, respectively. Data were presented as mean ± SD of three independent experiments. ^#^*p* < 0.05 compared with the control group; ^*^p < 0.05 compared with the LPS group; ^&^*p* < 0.05 compared with the self-repairing group.

After glycocalyx degradation, accelerating its restoration is very valuable for ARDS therapy. Therefore, we further analyzed the effect of Nef on glycocalyx restoration. In the experiments, Nef was given after LPS simulation to induce glycocalyx degradation (Figure 1A), The results showed that, compared with that in the self-repairing group, Nef post-treatment can accelerate endothelial glycocalyx compositions (HS and SDC-1) restoration both in LPS-induced ARDS mice (Figures 6A,B,E,F) and LPS-stimulated HUVECs (Figures 6C,D,G,H), indicating a promotion of Nef for glycocalyx reconstruction.

### Nef decreased MTROS production and suppressed oxidative damage in LPS-induced ARDS

**Figure 7:**
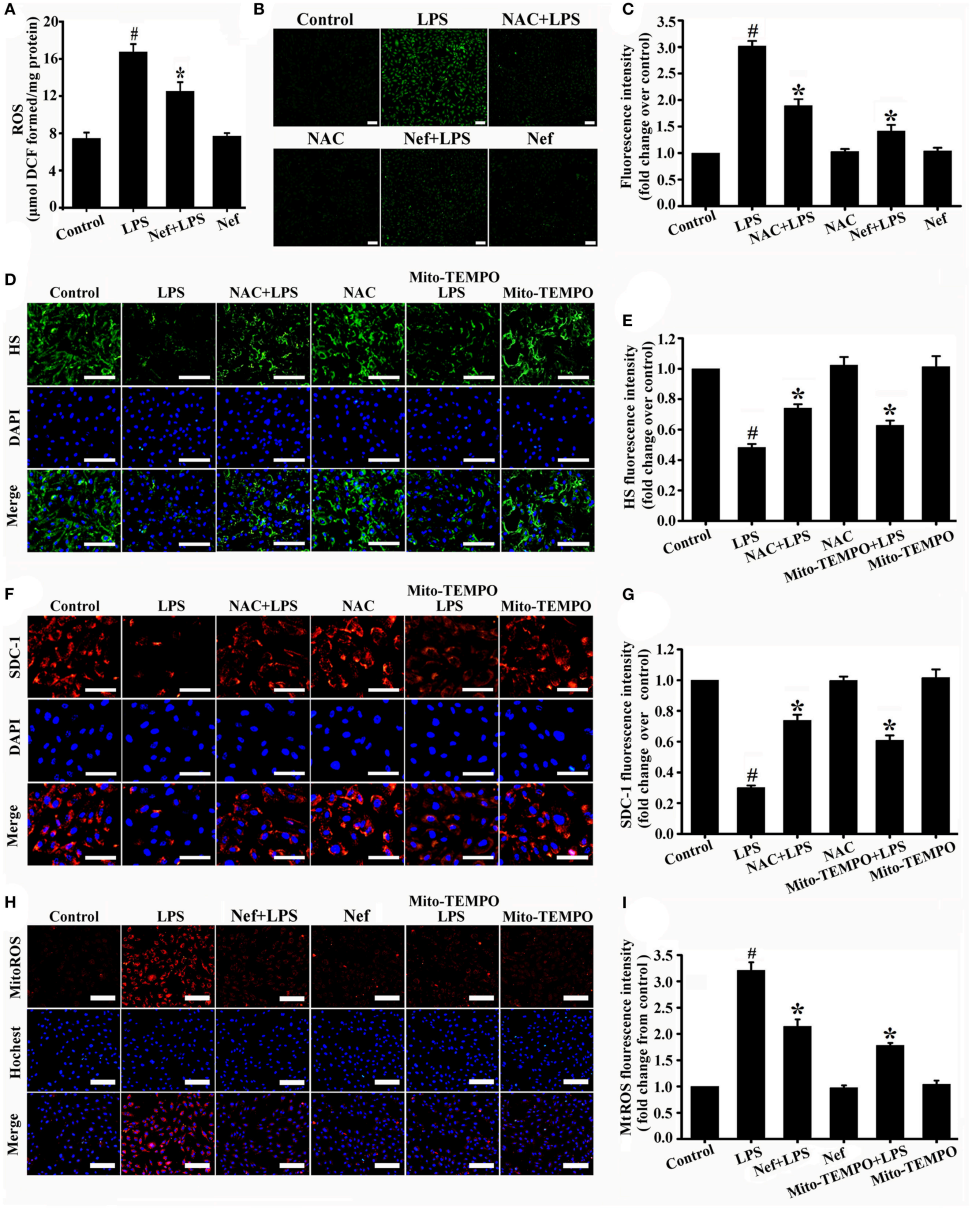
Effect of Nef on the production of intracellular ROS and mtROS in LPS-induced ARDS. **(A)** Intracellular ROS production was detected with DCFH-DA in mice lung tissue gavaged with Nef (20 mg/kg/day) for 7 days followed by LPS (20 mg/kg) for 6 h. **(B)** Representative fluorescence images of intracellular ROS production were detected with DCFH-DA in HUVECs pretreated with Nef (10 μM) for 1 h or NAC (1 mM) for 1 h followed by LPS (1 μg/mL) for 6 h. **(C)** Fluorescence intensity analyses of **(B)**. **(D)** Representative immunofluorescence images of HS component (green) and nuclei (blue) in control HUVECs and those treated with LPS (1 μg/mL) for 6 h, NAC (1 mM) for 1 h followed by LPS (1 μg/mL) for 6 h, NAC (1 mM) alone for 7 h, Mito-TEMPO (50 μM) for 1 h followed by LPS for 6 h, or Mito-TEMPO (50 μM) alone for 7 h. **(E)** Fluorescence intensity analyses of **(D)**. **(F)** Representative immunofluorescence images of SDC-1 component (red) and nuclei (blue) in control HUVECs and those treated with LPS (1 μg/mL) for 6 h, NAC (1 mM) for 1 h followed by LPS (1 μg/mL) for 6 h, NAC (1 mM) alone for 7 h, Mito-TEMPO (50 μM) for 1 h followed by LPS for 6 h, or Mito-TEMPO (50 μM) alone for 7 h. **(G)** Fluorescence intensity analyses of **(F)**. **(H)** Representative fluorescence images of mtROS (red) and nuclei (blue) in control HUVECs and those treated with LPS (1 μg/mL) for 6 h, Nef (10 μM) for 1 h followed by LPS (1 μg/mL) for 6 h, Nef (10 μM) alone for 7 h, Mito-TEMPO (50 μM) for 1 h followed by LPS (1 μg/mL) for 6 h, or Mito-TEMPO (50 μM) alone for 7 h. **(I)** Fluorescence intensity analyses of **(H)** Data were presented as were mean ± SD of three independent experiments. Scale bar: 100 μm. ^#^*p* < 0.05 compared with the control group; ^*^p < 0.05 compared with the LPS group.

ROS-induced oxidative damage has been shown to be one of the main reasons for glycocalyx degradation (van Golen et al., 2012; Singh et al., 2013; Chelazzi et al., 2015). In our experiments, LPS induces high intracellular ROS production in ARDS mice (*in vivo*) (Figure 7A) and HUVECs (*in vitro*) (Figures 7B,C). However, ROS scavenger NAC treatment would significantly reduce ROS production (Figures 7B,C) and decreased glycocalyx degradation (Figures 7D–G) simultaneously. These results further confirmed the relationship between ROS accumulation and endothelial glycocalyx degradation.

Previous study revealed that Nef has antioxidative functions (Jung et al., 2010; Peng et al., 2011; Baskaran et al., 2016). Such as Peng et al. have reported that Nef can improve the attenuated NO production in HUVECs induced by lysophosphatidylcholine via its antioxidant properties. Therefore, we determined whether the protective effect of Nef on the endothelial glycocalyx involves the suppression of ROS production in LPS-induced ARDS. As expected, the results showed that Nef can significantly reduce intracellular ROS production both in LPS-induced ARDS mice (Figure 7A) and LPS-stimulated HUVECs (Figures 7B,C).

ROS can be generated from different cellular sources. We further analyzed whether the mitochondria is the major source of intracellular ROS resulted in glycocalyx degradation. MtROS was highly generated in LPS-simulated HUVECs (Figures 7H,I). Mitochondria-targeted ROS scavenger Mito-TEMPO can significantly clear mtROS (Figures 7H,I) and simultaneously decrease glycocalyx shedding (Figures 7D–G). Notably, the Mito-TEMPO exerted the same degree of protective effect to endothelial glycocalyx as NAC (Figures 7D–G). This result suggested that mtROS is the major source of intracellular ROS resulting in glycocalyx degradation. As shown in Figures 7H,I, Nef pretreatment significantly decreased mtROS generation compared with that in the LPS group in LPS-stimulated HUVECs.

**Figure 8:**
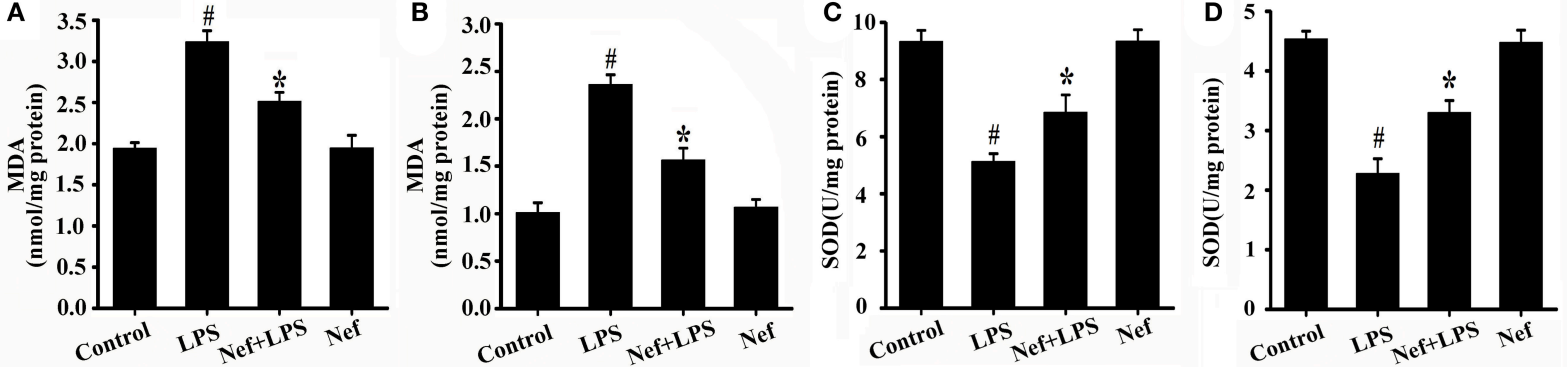
Effects of Nef on MDA production and SOD activity in LPS-induced ARDS. **(A)** MDA content was measured in mice lung tissue gavaged with 20 mg/kg/day Nef for 7 days and then with 20 mg/kg of LPS for 6 h. (*n* = 6 mice/group) **(B)** MDA content was measured in HUVECs pretreated with 10 μM Nef for 1 h and then with 1 μg/mL of LPS for 6 h. **(C)** SOD activity was determined in mice lung tissue that were gavaged with 20 mg/kg/day Nef for 7 days, followed by 20 mg/kg of LPS for 6 h. **(D)** SOD activity was determined in HUVECs pretreated with 10 μM Nef for 1 h, followed by pretreatment with 1 μg/mL of LPS for 6 h. Data were presented as were mean ± SD of three independent experiments. ^#^*p* < 0.05 compared with the control group; ^*^p < 0.05 compared with the LPS group.

The effect of Nef on oxidative damage in LPS-induced ARDS was evaluated by measuring the production of MDA, a maker of lipid peroxidation. As shown in Figures 8A,B, the MDA content was reduced in the Nef treatment group compared with that in the LPS group in LPS-induced ARDS mice (Figure 8A) and LPS-stimulated HUVECs (Figure 8B).

Intracellular redox imbalance is an important cause of LPS-induced oxidative stress (Wang G. et al., 2016; El Kamouni et al., 2017). Furthermore, the effect of neferine (Nef) on the antioxidative enzyme superoxide dismutase (SOD) was also determined. As shown in Figure 8, SOD activity increased in the Nef treatment group in contrast to that in the LPS group in the LPS-induced ARDS mice (Figure 8C) and LPS-stimulated HUVECs (Figure 8D), suggesting that Nef contributed to the restoration of the balance between oxidation and antioxidation.

Taken together, these results indicated that the protective effect of Nef on the endothelial glycocalyx may be initiated by suppressing mtROS production and in turn decreasing oxidative damage.

**Figure 9:**
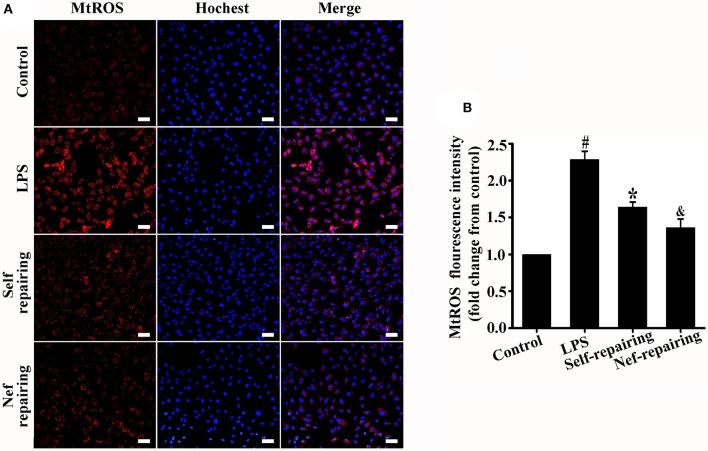
Measurement of mtROS in glycocalyx restorated endothelial cells after Nef treatment. **(A)** Representative fluorescence images of mtROS (red) and nuclei (blue) in control HUVECs and those treated with LPS for 6 h, LPS for 6 h followed by complete medium without LPS for 12 h, or LPS for 6 h followed by Nef for 12 h. **(B)** Fluorescence intensity analyses of **(A)**. Data were presented as were mean ± SD of three independent experiments. Images were taken at 100 × magnification, scale bar: 100 μm. ^#^*p* < 0.05 compared with the control group; ^*^p < 0.05 compared with the LPS group; ^&^*p* < 0.05 compared with the self-repairing group.

The level of mtROS was detected in endothelial cells with glycocalyx restoration. As shown in Figure 9, the mtROS level in Nef-treated endothelial cells (Nef-repairing group) was lower than that without treatment (self-repairing group). This result suggested that Nef can promote glycocalyx reconstruction by accelerating mtROS elimination of glycocalyx damaged endothelial cells in LPS-induced ARDS.

## Discussion

The endothelial glycocalyx serves as the primary physical barrier and interface between the blood and the vessel wall, which is damaged in ARDS. In this study, we found that Nef can protect against endothelial glycocalyx degradation and reduce lung-capillary permeability in LPS-induced ARDS. Although Nef has been shown to exert protective effects in several diseases by affecting signal transduction, cell proliferation, cell apoptosis and cell autophagy (Zhang et al., 2012; Guan et al., 2014; Eid and Abdel-Rehim, 2017; Sivalingam et al., 2017; Priya et al., 2018), to our knowledge, this study has first revealed its protective role in endothelial glycocalyx.

Oxidative stress has been shown to be an important factor for endothelial glycocalyx degradation. We showed that treatment with ROS scavenger NAC and mitochondria-targeted antioxidant Mito-TEMPO can reduce LPS-induced endothelial glycocalyx damage in ARDS and further highlighted that decreasing oxidative damage is an effective way to alleviate glycocalyx shedding. Further study showed that Nef can effectively decrease intracellular ROS and mtROS production and suppress oxidative damage in LPS-induced ARDS. These results indicated that suppressing mtROS-induced oxidative damage might be one of the main mechanisms for the protective effect of Nef on the endothelial glycocalyx in LPS-induced ARDS.

Additionally, enzymatic degradation is another important mechanism for endothelial glycocalyx degradation. As such, endogenous HS-specific glucuronidase heparanase (Schmidt et al., 2012; Lukasz et al., 2017) and matrix metalloproteinase (MMPs) (Ramnath et al., 2014; Zeng et al., 2014) are important endothelial glycocalyx degradation enzymes. Notably, a recent study reported that combined treatment of Nef and cisplatin can significantly inhibit MMP-2 enzyme activity in human lung cancer cells, although treatment with Nef alone did not show significant effects (Sivalingam et al., 2017). Further investigations are needed to determine whether the endothelial glycocalyx protective effect of Nef is also associated with inhibition of enzymatic degradation.

Inflammatory responses play a critical role in the initiation and development of ARDS. In this study, we found that the Nef treatment can down-regulate cytokine production and inhibit NF-κB activation, indicating that Nef also ameliorates LPS-induced ARDS through its anti-inflammatory effects. Inflammatory responses also have a close relationship with the modulation of endothelial glycocalyx structure. A large number of substances causing damage to glycocalyx are induced or activated during inflammatory conditions. These substances include ROS, MMPs, heparanase, and neutrophil elastase (Schmidt et al., 2012; Kolárová et al., 2014; Martin et al., 2016). Therefore, reducing inflammatory response after Nef treatment decreases the rate of endothelial cell glycocalyx damage.

Achieving rapid restoration of the damaged endothelial glycocalyx will contribute to the treatment of ARDS. Recent reports showed that sepsis causing vascular injury may not only induce endothelial glycocalyx destruction but also impair fibroblast growth factor receptor 1/exostosin-1-mediated glycocalyx reconstitution (Yang et al., 2017). Song JW et al. reported that sulodexide, which is a heparan sulfate mimetic and resistant to heparanase, can improve the restoration of endothelial glycocalyx in sepsis (Song et al., 2017). In the present study, we showed that Nef also promoted endothelial glycocalyx repair in LPS-induced ARDS *in vivo* and *in vitro*. Further study suggested that Nef can promote the removal of mtROS in glycocalyx damaged endothelial cells, which in turn accelerate the recovery of endothelial glycocalyx.

Notably, as the shortcoming of this paper, the related pharmacokinetic study on Nef in the body of mice was not conducted after using the drug in LPS-induced ARDS mouse model, for example, to detect processes such as absorption, distribution, metabolism, and excretion. A detailed understanding of the biological half-life of Nef and its bioavailability will aid in its application in treatment of ARDS and other diseases.

In conclusion, our study showed that Nef can alleviate endothelial glycocalyx degradation and promote endothelial glycocalyx repair by suppressing mtROS-induced oxidative damage in LPS-induced ARDS. The results of the study suggested the potential use of Nef as a therapeutic agent in ARDS induced by gram-negative bacterial infections and increased our knowledge about the mechanisms of endothelial glycocalyx protection/restoration.

## Author contributions

X-YL, H-XX, J-KL, and DZ performed the mainly experiments, analyzed data and wrote the first draft. J-KL contributed to animal experiments and data analysis. X-HM and L-NH contributed to cell *in-vitro* experiments, figures drawing and design. J-HL contribute to drafting and critically revising the manuscript. X-YL and X-ZW contributed to design of the work, analysis of data, revising and (final) approval of the manuscript.

### Conflict of interest statement

The authors declare that the research was conducted in the absence of any commercial or financial relationships that could be construed as a potential conflict of interest.
