# Supplementary figures and images for: Neferine Protects Endothelial Glycocalyx via Mitochondrial ROS in Lipopolysaccharide-Induced Acute Respiratory Distress Syndrome
Source: Front Physiol. 2018 Feb 22;9:102. doi: 10.3389/fphys.2018.00102 (PMC5826949; doi:10.3389/fphys.2018.00102)

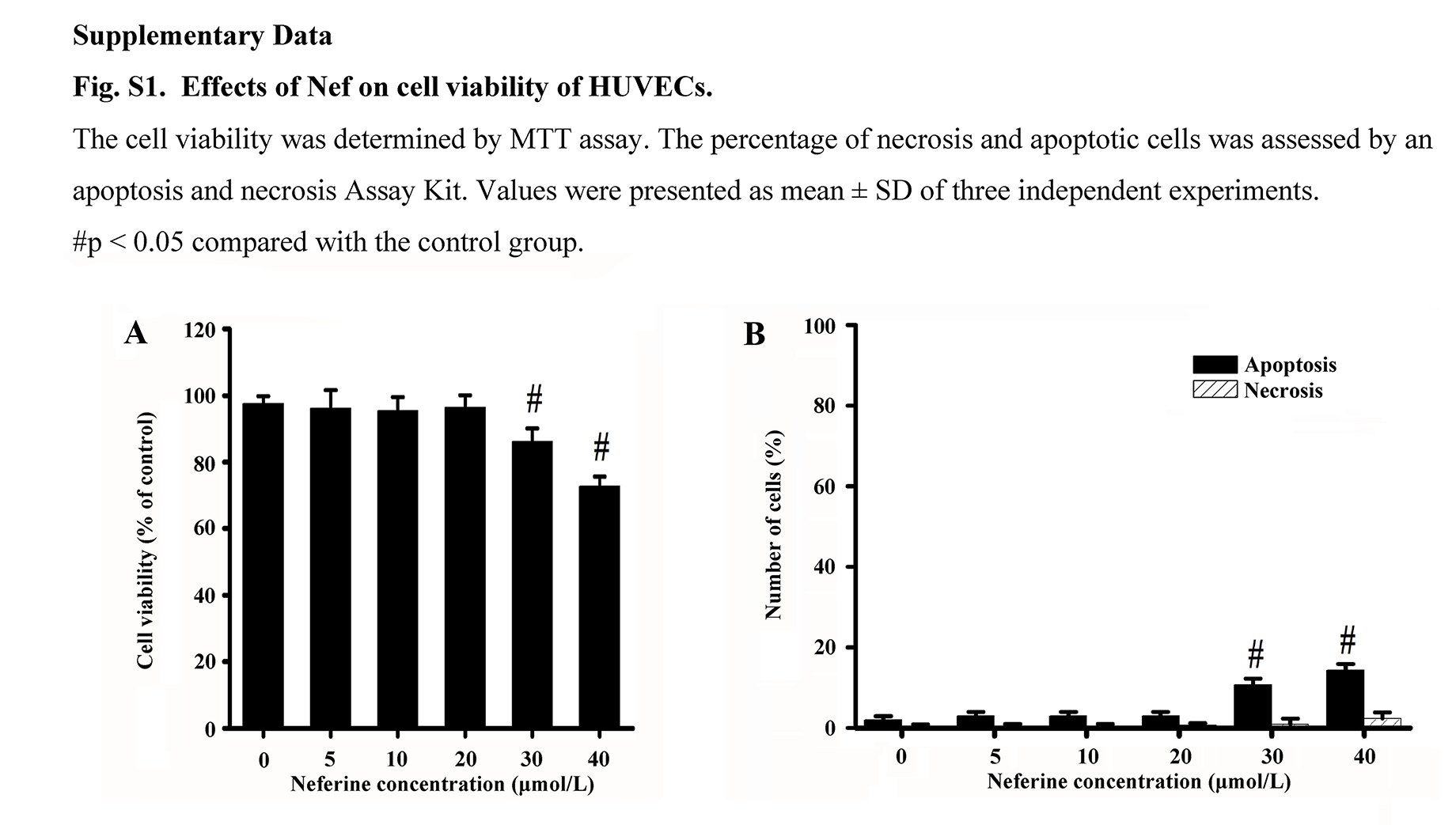

Supplement: Supplementary file 1 [file Image1.TIF]
